# Relationship between bone density and bone metabolism in adolescent idiopathic scoliosis (AIS)

**DOI:** 10.1186/1748-7161-8-S2-O4

**Published:** 2013-09-18

**Authors:** Yoichi Aota, Ko Ishida, Nobuyuki Tanabe, Tomoyuki Saito

**Affiliations:** 1 Department of Orthopaedic Surgery, Yokohama City University, Yokohama, Japan

## Purpose

Although osteopenia is often associated with AIS, studies on bone metabolism in relation to AIS have not yielded clear results. To characterize bone metabolism in AIS patients, a cross sectional study assessing bone metabolism and bone density was performed.

## Methods

Using dual–energy X ray absorptiometry and bone metabolism markers (bone formation marker; BAP, bone resorption marker; TRAP5b), the bone mineral density of lumbar and bilateral proximal femurs were studied in 41 AIS patients aged 10 to 20 years old, with a mean of 15.2±5.9 years old. Divided into two groups by levels of bone resorption marker (TRAP5b), BMD, BMI and age of menarche were compared statistically in each group.

## Results

Among the AIS patients studied, osteopenia (-1 standard deviations to -2 standard deviations) was found in 34.1% of the patients and osteoporosis (below -2 standard deviations) was found in 24.4% of the patients. In 39 AIS patients (95.2%), BAP values were within normal range. On the other hand, TRAP5b values were significantly high in 65.9% of the patients. In high levels of the TRAP5b group, BMD values of the lumbar spine and right femoral neck were significantly lower than those of the TRAP5b group.

## Conclusions and discussion

The bone resorption marker was high in 65.9% of the AIS patients, and high bone resorption in bone metabolism was found to be a possible cause of low bone mineral density in patients with AIS.
